# The Molecular Mechanisms of Oleanane Aldehyde-β-enone Cytotoxicity against Doxorubicin-Resistant Cancer Cells

**DOI:** 10.3390/biology12030415

**Published:** 2023-03-08

**Authors:** Natalia Moiseeva, Daria Eroshenko, Lidia Laletina, Ekaterina Rybalkina, Olga Susova, Aida Karamysheva, Irina Tolmacheva, Mikhail Nazarov, Victoria Grishko

**Affiliations:** 1 The N.N. Blokhin National Medical Research Center of Oncology, Health Ministry of Russia, 115478 Moscow, Russia; 2 Institute of Technical Chemistry, Perm Federal Scientific Centre, Ural Branch, Russian Academy of Science, 614013 Perm, Russia

**Keywords:** triterpenoids, cytotoxicity, drug resistance, apoptosis, cell cycle, caspases

## Abstract

**Simple Summary:**

Currently, the main reason for the ineffectiveness of systemic chemotherapy is the formation of cancer cells with the phenotype of multiple drug resistance (MDR), which are able to survive at high doses of chemotherapy drugs. One of the MDR mechanisms involves the overproduction of proteins of the ABC transporter family, including P-glycoprotein, which are responsible for the transport of a wide range of drugs from the cell. This paper focuses on a new derivative of the natural triterpenoid betulin, oleanane aldehyde-β-enone (**OA**), having low toxicity toward normal cells and cytotoxicity toward cancer cells and their doxorubicin-resistant variants. The calculations and experimental methods revealed that this compound is not released from cells like most known drugs. The analysis of molecular cellular targets showed that the mechanism of **OA** toxic action is associated with the activation of the external and/or internal pathways of the apoptotic death of cancer cells and their drug-resistant variants. Thus, **OA**, as a highly cytotoxic compound toward doxorubicin-resistant cancer cells, can be a promising candidate for drug development.

**Abstract:**

Oleanane aldehyde-β-enone (**OA**), being the semi-synthetic derivative of the triterpenoid betulin, effectively inhibits the proliferation of HBL-100 and K562 cancer cells (IC_50_ 0.47–0.53 µM), as well as the proliferation of their resistant subclones with high P-gp expression HBL-100/Dox, K562/i-S9 and K562/i-S9_Dox (IC_50_ 0.45−1.24 µM). A molecular docking study, rhodamine efflux test, synergistic test with Dox, and ABC transporter gene expression were used to investigate the ability of **OA** to act as a P-gp substrate or inhibitor against Dox-resistant cells. We noted a trend toward a decrease in *ABCB1*, *ABCC1* and *ABCG2* expression in HBL-100 cells treated with **OA**. The in silico and in vitro methods suggested that **OA** is neither a direct inhibitor nor a competitive substrate of P-gp in overexpressing P-gp cancer cells. Thus, **OA** is able to overcome cellular resistance and can accumulate in Dox-resistant cells to realize toxic effects. The set of experiments suggested that **OA** toxic action can be attributed to activating intrinsic/extrinsic or only intrinsic apoptosis pathways in Dox-sensitive and Dox-resistant cancer cells, respectively. The cytotoxicity of **OA** in resistant cells is likely mediated by a mitochondrial cell death pathway, as demonstrated by positive staining with Annexin V–FITC, an increasing number of cells in the subG0/G1 phase, reactive oxygen species generation, mitochondrial dysfunction, cytochrome *c* migration and caspases-9,-6 activation.

## 1. Introduction

Despite significant progress in cancer therapy, including traditional methods of surgery and radiation therapy as well as modern immunotherapy and gene therapy, chemotherapy is still the most commonly used therapy in cancer treatment. There are three major morphological types of cancer cell death, including apoptosis, autophagy and necrosis [1]. Apoptosis, the cell’s natural mechanism for programmed cell death, is a main target for anticancer therapy [2]. Concurrently, cancer chemotherapy is well known to often cause multi-drug resistance (MDR) in cancer cells, thus diminishing the efficacy of treatment [3]. This is why anti-drug-reducing agents are becoming increasingly important in the search for new therapies [4].

Various mechanisms could be responsible for MDR, such as certain genetic factors, increased DNA repair capacity or peculiarities of the metabolism, but the most important contributing factor is the efflux of drugs from cancer cells [5]. ATP-binding cassette (ABC) proteins, including ATP-binding cassette subfamily B member 1 (ABCB1) or P-glycoprotein (P-gp), Breast Cancer Resistance Protein (BCRP/ABCG2) and Multidrug-Resistance Protein 1 (MRP1/ABCG1), play a crucial role in the transport of drugs from the cells and are often activated as a result of chemotherapy [6]. There exist several possibilities for a compound to overcome MDR caused by ABC-transporter protein activation via the inhibition of P-gp-mediated drug efflux or the suppression of P-gp expression [7]. A compound which could interact with the active site of ABC-transporter proteins belongs to the substrate of MDR proteins and competes for the active site of MDR proteins, when applied in combination with another substrate. Conversely, a compound belongs to an inhibitor if it can disturb the protein structure by binding both the active site and another domain. The capability of novel, potent and nontoxic compounds to overcome MDR could be an important reason for considering it as a promising one for further development as a new chemotherapeutic drug [8]. 

Naturally occurring compounds have been an important source of new therapeutically active agents for cancer treatment, including MDR inhibitors [9,10,11]. Among phytochemicals, the pentacyclic triterpenoids of the lupane, oleanane and ursane types are characterized by a wide variety of structures and a broad spectrum of biological activities, but these triterpenoids suffer from poor aqueous solubility, low bioavailability and limited intracellular accumulation capacity, which are unfavorable for drug development [12]. In order to improve bioavailability and efficiently enhance the anticancer activity (often without causing toxicity toward normal cells) of these compounds, different approaches have been performed, including simple synthetic transformations [13,14]. 

The mechanisms of the anticancer action of triterpenic derivatives are often multi-targeted and mainly associated with DNA polymerase inhibition, the regulation of apoptosis, a change in signal transductions, interference with angiogenesis and dedifferentiation, antiproliferative activity and metastasis inhibition [15]. Pentacyclic triterpenoids with cell-proliferation-inhibitory properties most often act as inducers of intrinsic and extrinsic apoptotic signaling pathways [16]. Among the multiple molecular targets and cell proliferation regulatory pathways of triterpenic derivatives, the ability of triterpenoids to reverse various potential mechanisms contributing to the MDR of cancer cells has been increasingly reported in recent years [17,18,19]. Previously, we demonstrated the ability of the semi-synthetic derivatives of the triterpenoid betulin to effectively suppress the expression of ABC genes and the transport functions of the P-gp protein [20]. 

Recently, on the basis of betulin, we synthesized a novel derivative, oleanane aldehyde-β-enone (**OA**), which showed cytotoxic action against human cancer cells of different origin [21]. Continuing the study of the biological properties of oleanane aldehyde-β-enone, we report the cytotoxic activity of **OA** against several new cancer cell lines, including Dox resistance, owing to its overexpression of the P-gp protein. To understand the possible mechanism of the antiproliferative effect of **OA** against Dox-resistant cancer cells, we evaluated P-gp functional activity, ABC transporter gene expression, apoptotic activity and cell cycle alteration in Dox-resistant cells treated with **OA**. A molecular docking study and rhodamine efflux test were carried out to investigate the ability of **OA** to act as a P-gp substrate or inhibitor. In addition, the proapoptotic effect of the semi-synthetic triterpenoid in Dox-sensitive and Dox-resistant cancer cells was investigated for the first time.

## 2. Materials and Methods

### 2.1. Drugs and Reagents

Oleanane aldehyde-β-enone (**OA**) used in the present investigation was synthesized from the available triterpenoid betulin, as previously described [21]. The NMR spectral data of **OA** coincided with the description.

*2-Formyl-19β,28-epoxy-18α-olean-1(2)-en-3-one* (**OA**). Yield: 77%. Mp: 173.6 °C (pet. ether/ethyl acetate 7:1). [α]D^23^ −9.7 (c 0.5; CHCl_3_); Ref. [21]: Yield (80%). Mp: 172–176 °C. [α]D^21^ −9.6 (c 0.6; CHCl_3_). 

### 2.2. Cell Line and Culture Conditions

The following cell lines were used in this study: HBL-100 (human mammary epithelial cells immortalized by SV-40 virus, which, due to long-term cultivation, became tumorigenic) [22], HCT-116 (wild-type colon cancer cells), HCT 116 p53^−/−^ (p53 knocked-out colon cancer cells), K562 (chronic myelogenous leukemia), MDA-MB-231 (human breast cancer), MDA-MB-453 (human breast cancer) and MCF10A (mammary epithelial cell line). The HBL-100, HCT-116, HCT 116 p53^−/−^ and K562 cells were cultivated in RPMI1640 medium (PanEko, Moscow, Russia) with the addition of 10% fetal bovine serum (HyClone, Logan, UT, USA) and gentamycin (40 µg/mL). MDA-MB-231 and MDA-MB-453 were cultivated in DMEM medium (PanEko, Moscow, Russia) with the addition of 10% fetal bovine serum (Biosera, Nuaillé, France), 2 mM L–glutamine (Gibco, New York, NY, USA) and gentamycin (40 µg/mL). The MCF10A cells were cultivated in DMEM/F12 medium (PanEko, Moscow, Russia) with 5% horse serum (HyClone, Logan, UT, USA), 2 mM L–glutamine (PanEko, Moscow, Russia), 20 ng/mL epidermal growth factor (Sigma, St Louis, MO, USA), 10 mg/mL insulin (Sigma, St Louis, MO, USA), 500 nM hydrochortisone (Sigma, St Louis, MO, USA) and 40 µg/mL gentamycin. 

### 2.3. P-Glycoprotein Overexpressing Cell Cultures and Culture Conditions

Several cell cultures with a multidrug-resistance phenotype developed, owing to the overexpression of P-glycoprotein. The K562/i-S9 subclone with the functionally active P-gp was obtained as the result of stable *ABCB1* (*MDR1*) gene transfection [23]; the HBL-100/Dox cell line and K562/iS9_Dox subclone were derived from HBL-100 and K562/iS9 cells, respectively, after continuing cell selection in the presence of a rising concentration of Doxorubicin (Dox; TEVA, Tel-Aviv, Israel). P-gp expression in the obtained sublines was previously assessed; in HBL-100 and K562 cells, P-gp is not expressed; in the K562/i-S9 subline, 75% of cells have a high P-gp expression; and in HBL-100/Dox and K562/iS9_Dox, about 95% of cells have highly expressed P-gp [24]. The HBL-100/Dox, K562/i-S9 and K562/iS9_Dox cells were cultivated in RPMI1640 medium (PanEko, Moscow, Russia) with the addition of 10% fetal bovine serum (Biosera, Nuaillé, France) and gentamycin (40 µg/mL).

### 2.4. MTT Cell Viability Assay

Cell viability was evaluated via an MTT cell viability assay [25]. MTT (3-[4,5-dimethylthiazol-2-yl]-2,5-diphenyltetrazolium bromide) was used as a colorimetric substrate for measuring cell viability. Non-viable cells, with altered cellular redox activity, were unable to reduce the MTT dye. The cells (5000 cells/well in 100 μL of medium for adherent cell lines and 25,000 cells/well in 150 μL of medium for suspension cell lines) were incubated in 96-well plates in a 5% CO_2_ incubator at 37°C. Compound **OA** and Dox were dissolved in DMSO (AppliChem, Council Bluffs, IA, USA) to a concentration 10.0 mM. After cultivating the cells in a pure medium for 24 h, Dox and **OA** were added to the cells at ten different concentrations ranging from 0.2 to 100.0 μM. The cells were cultivated with the compounds for 72 h; then, MTT (5 mg/mL) was added (20 μL/well). After 2 h of incubation, the medium with the compounds was removed, and the resulting formazan precipitate was dissolved in 100 μL of DMSO. The absorbance in the relevant wells was measured at 540 nm using a microplate photometer Multiskan FC (Thermo Scientific, Waltham, MA, USA). The IC_50_ value (the concentration of a substance causing 50% of cell death) was determined graphically based on the results of the MTT reaction.

### 2.5. P-Gp Substrate and P-Gp Inhibitor Prediction

We used four online services to predict whether triterpenoid **OA** is a P-gp substrate or an inhibitor. First, the new version of admetSAR (version 2.0), mainly focusing on the in silico prediction of chemical ADMET properties, is available at https://admetmesh.scbdd.com/ (accessed on 1 February 2023) [26].

ADMETlab 2.0 is an extended version of the widely used ADMETlab for systematically evaluating ADMET properties, as well as some physicochemical properties and medicinal chemistry friendliness [27]. Predictions of a substance as being a P-gp substrate or an inhibitor can be freely accessed in the Absorption section at http://lmmd.ecust.edu.cn/admetsar2 (accessed on 1 February 2023).

A novel approach of the Bio21 Molecular Science and Biotechnology Institute for predicting pharmacokinetic properties, called pkCSM, which relies on graph-based signatures, is provided online at http://biosig.unimelb.edu.au/pkcsm/ (accessed on 1 February 2023). These encoded distance patterns between atoms are used to represent small molecules and to train predictive models [28].

The PgpRules prediction models, based on the classification and regression of a tree algorithm, were provided by Wang et al. [29] as a web application, which can be freely accessed at https://pgprules.cmdm.tw/ (accessed on 1 February 2023). 

### 2.6. Molecular Docking of P-Gp

The molecular docking studies were carried out on a laptop PC with an installed Intel^®^ Core i3-6100 QM CPU with 3.70 GHz and RAM 8 GB operating under the Windows 8 Professional OS. Briefly, the 3D structures of human P-gp with the bound substrate (PDB ID: 6QXE) and inhibitor (PDB ID: 6QEE) were loaded into GOLD, version 5.0.1 (CCDC Software, London, UK) with the addition of all the hydrogen atoms. All the types of atoms, charges and bond hybridization were carefully checked. The structure of **OA** was optimized using the MM2 force field integrated in the ChemBio3D Ultra 14.0 software (Perkin Elmer, Waltham, MA, USA). The docking was performed using the GOLD wizard (400 docking poses), and the Chemscore values were determined. The conformation of the ligand with the highest Chemscore value was selected as the best one and was then compared with the conformation of conventional and bound P-gp substrates (verapamil and paclitaxel) and inhibitors (tariquidar and zosuquidar) with Maestro 11.6 software (Schrödinger LLC, New York, NY, USA).

### 2.7. Evaluation of Cell Sensitization to Doxorubicin

The combined effect of **OA** and Dox was assessed using the MTT test. The cells were seeded into a 96-well plate (5000 cells/well in 100 µL of medium for adherent cell lines, and 25,000 cells/well in 150 μL of medium for suspended cell lines). After 24 h of incubation in a 5% CO_2_ incubator at 37 °C, **OA** was added to the wells at a concentration of 0.2 μM (for HBL-100, K562 and K562/i-S9) and 0.6 μM (for HBL-100/Dox). This concentration was non-toxic to all the cells, and cell survival exceeded 90%. Concurrently with **OA**, Dox at different concentrations was added to the wells. The cells were incubated for another 72 h. After that, the MTT reagent was added to the wells, and 2 h later, all manipulations were performed as described above. The IC_50_ values were compared for Dox alone and when combined with **OA**.

### 2.8. Evaluation of P-Gp Functional Activity

The functional activity of P-gp was evaluated using a previously described method [30]. The K562/i-S9 cells were incubated for 20 min in a culture medium containing 5.0 μg/mL Rhodamine 123 (Rh123; Sigma-Aldrich, St. Louis, MO, USA). After incubation, the cells were washed twice and divided into several fractions (5 × 10^5^ cells per point). One fraction was incubated in a pure medium, and the others were incubated with the addition of 1.0 μM and 20.0 μM of the investigated substance. The well-known P-gp inhibitor verapamil (TEVA, Tel-Aviv, Israel) served as a reference compound. Incubation was carried out in culture medium RPMI1640 without FBS at 37 °C for 30 min. Cell fluorescence was evaluated via a FACScan flow cytometer (Becton Dickinson, Franklin Lakes, NJ, USA). The results were analyzed using FlowJo software (ver. X 10.0.7r2, FlowJo Software, San Jose, CA, USA) and were represented with the geometric mean fluorescence intensity (gMFI) and MAF coefficient [31], which was calculated using the following formula: MAF = (Mean (inh) − Mean (free))/Mean (inh), where MAF is the MDR Activity Functional, Mean (free) is the mean value of cell fluorescence without the inhibitor, and Mean (inh) is the mean value for cell fluorescence with the inhibitor.

### 2.9. **OA** Effect on ABC Transporter (ABCB1, ABCC1 and ABCG2) Gene Expression 

#### 2.9.1. Cell Line and Culture Conditions

The effect of **OA** on the expression of ABC-transporters (*ABCB1*, *ABCC1* and *ABCG2*) was studied in pairs of cell lines K562 and K562/i-S9, as well as HBL-100 and HBL-100/Dox. The cells were seeded 2 × 10^6^/dish, and **OA** and Dox were added immediately to suspension cultures and on the next day to adherent cultures. Concentrations of substances were used corresponding to their IC_50_ values, the cells were incubated for 48 h, and then RNA was isolated.

#### 2.9.2. RNA Isolation, Reverse Transcription and Real-Time PCR 

PureZOL RNA isolation reagent (Bio-Rad Laboratories, Hercules, CA, USA) was used for RNA isolation, according to the manufacturer’s protocol. RNA concentration was measured using a NanoVue Plus spectrophotometer (GE Healthcare, Chicago, IL, USA) and was calculated on the basis of its optical density at 260 nm. The integrity of RNA was checked with 1% agarose gel electrophoresis containing 0.01% ethidium bromide. RNA reverse transcription was carried out with random hexanucleotide primers and M-MLV reverse transcriptase (Thermo Fisher Scientific, Waltham, MA, USA). Then, real-time PCR was performed on a CFX Connect Real-Time PCR Detection System (Bio-Rad Laboratories, Hercules, CA, USA). The amplification steps were as follows: 95 °C—3:00 min, (95 °C—0:10 min, 60 °C—00:10 min, and 72 °C—00:30 min) with 39 cycles and a melt curve of 65–95°C. The housekeeping gene RPL0 was used to normalize gene expression data. The primers for real-time PCR are given in Table S1.

The relative expression level for each gene was calculated as [∆C_t_ = C_t_ (investigated gene) − C_t_ (RLP0), with C_t_ being the minimal baseline cycle in the exponential phase of the amplification curve]. The relative difference in gene expression levels was determined as 2^−ΔΔCt^, where ΔΔC_t_ = ΔC_t_ (a treated sample) − ΔC_t_ (an untreated control).

### 2.10. Annexin V-FITC/PI Assay 

This assay was conducted according to the manufacturer’s protocol (Annexin V-FITC/PI Apoptosis Detection Kit 1; Elabscience, Houston, TX, USA). Briefly, the cells were seeded into a 6-well plate and incubated for 24 h at 37 °C in a humidified atmosphere containing 5% CO_2_. The medium in each well was subsequently replaced with a fresh medium containing IC_50_ and 2 × IC_50_ values of **OA**. Dox was used as a positive control of apoptosis alteration. After a 16 h incubation period (for adherent cultures) or 24 h (for suspension cultures), all the detached/dead and viable cells were collected. The cells were then washed and re-suspended with PBS. The harvested cells were stained with Annexin V for 30 min before being treated with PI and analyzed with a FACScan flow cytometer (Becton Dickinson, Franklin Lakes, NJ, USA) for suspension cultures, or a Cytoflex S flow cytometer (Beckman Coulter, San Jose, CA, USA) for adherent cultures. The results were analyzed using FlowJo software (ver. X 10.0.7r2, FlowJo Software, San Jose, CA, USA). 

### 2.11. Cell Cycle Analysis 

The HBL-100 and HBL-100/Dox cells were seeded into a 6-well plate and incubated for 24 h at 37 °C in a humidified atmosphere containing 5% CO_2_. The medium in each well was replaced with a fresh medium containing the IC_50_ values of the test compounds. Dox was used as a positive control of cell cycle alteration. After a 16 h incubation period, all the detached/dead and viable cells were collected, washed with cold PBS and re-suspended in 300 μL of cold PBS before being treated with 4.5 mL of cold 70% ethanol. The cells were then incubated for 2 h at 4 °C. At the end of the incubation period, the cells were centrifuged (Model 5417 R, Eppendorf, Hamburg, Germany) at 1500× *g* for 5 min, and the resulting pellet was washed with cold PBS and re-suspended in 300 µL of PBS. The cells were incubated with 1.0 µg/mL of DAPI (Sigma, St Louis, MO, USA) on ice for 30 min in darkness. The distribution of cells was immediately analyzed via flow cytometry using a Cytoflex S flow cytometer (Beckman Coulter, San Jose, CA, USA) and with ModFit software (ver. 3.2.1, ModFit software, Bedford, MA, USA).

### 2.12. Interaction of Compounds with DNA

Different concentrations of the test compounds (2.5, 10.0, 20.0, 50.0 and 100.0 μM) were incubated with 0.1 μg of plasmid DNApHOT1 (TopoGen, Buena Vista, CO, USA) at 37 °C for 30 min in 10.0 mM Tris-HCl (pH 7.9). Carboplatin (TEVA, Tel Aviv, Israel) was used as a positive control of the retardation of DNA migration. The reaction products were separated in 1% agarose gel electrophoresis under a maximal voltage of 1.8 V/cm for 5 h. The gel was then stained with 0.5 μg/mL ethidium bromide. The DNA contained in the gel was visualized via UV fluorescence with a ChemiDoc MP Imaging system (Bio-Rad Laboratories, Hercules, CA, USA) at a wavelength of 254 nm. 

### 2.13. Inhibition of Topoisomerase I Catalytic Activity 

The modulation of Topoisomerase I (Topo I) activity in vitro was studied using the Topo I Drug Screening kit (TopoGen, Buena Vista, CO, USA). The studied compound at different concentrations (2.5, 10.0, 20.0, 50.0 and 100.0 μM) or camptothecin (CPT; Sigma-Aldrich, St. Louis, MO, USA) at different concentrations (100.0 and 200.0 μM) and 3.5 units of purified recombinant human Topo I were incubated with 0.26 μg of supercoiled pHOT1 plasmid DNA (TopoGen, Buena Vista, CO, USA) in a reaction buffer (10 mM Tris-HCl, pH 7.9, 1 mM EDTA, 0.15 M NaCl, 0.1% BSA, 0.1 mM spermidine, 5% glycerol). The mixture was incubated for 30 min at 37 °C, and the reaction was discontinued by adding SDS to reach a final concentration of 1%. Next, the reaction mixture was treated with proteinase K at a final concentration of 50 μg/mL for 30–60 min at 37 °C. The reaction products were separated electrophoretically in 0.9% agarose gel with TAE buffer (2.0 M Tris base, 0.05 M EDTA, 1.56 M acetic acid) under a maximal voltage of 3–4 V/cm. The gel was then stained with 0.5 μg/mL ethidium bromide. The DNA in the gel was visualized via UV fluorescence with the ChemiDoc MP Imaging system (Bio-Rad Laboratories, Hercules, CA, USA) at wavelengths in the range of 240 to 360 nm. 

### 2.14. Confocal Fluorescent Microscopy 

The HBL-100 and HBL-100/Dox cells were seeded on confocal dishes with glass bottoms and incubated for 24 h at 37 °C in a humidified atmosphere containing 5% CO_2_. The max cell density was 1 × 10^5^ cells/cm^2^. The medium was replaced with fresh medium containing 0.5 or 1.0 μM of the **OA** compound or 0.5 or 25.0 μM Dox for HBL-100 and HBL-100/Dox cells, respectively. DNA marker Hoechst 33342 Ready Flow Reagent was used as a stock solution (Invitrogen, ThermoFisher Scientific, Waltham, MA, USA). Mitochondrial marker LumiTracker Mito TMRE (tetramethylrhodamine, ethyl ester; Lumiprobe, Moscow, Russia) was dissolved in DMSO as a 1.0 mM stock solution. The marker of reactive oxygen species, H_2_DCFDA (Lumiprobe, Moscow, Russia), was dissolved in DMSO as a 100.0 μM stock solution. After a 16 h incubation period, the cells were incubated with a serum-free culture medium containing Hoechst 33342 dye (final concentration of 1.0 μM), LumiTracker Mito TMRE dye (final concentration of 1.0 μM) and H_2_DCFDA dye (final concentration of 1.0 μM) for 30 min at 37 °C in darkness. Fluorescence at 420 nm (Hoechst 33342), at 510 nm (TMRE) and at 575 nm (H_2_DCFDA) of the test cultures (including controls) was analyzed immediately. The cells were observed and photographed under a fluorescence microscope, Olympus CKX 53 (Tokyo, Japan), in darkness. 

### 2.15. Western Blotting

The HBL-100 and HBL-100/Dox cells were cultured in a 6-well plate at a density of 2 × 10^6^ cells/well. After treatment with IC_50_ and 2 × IC_50_ concentrations of **OA** for 16 h, all the detached/dead and viable cells were harvested and lysed in the Mitochondria Isolation Kit for Cultured Cells (Thermo Fisher Scientific, Waltham, MA, USA). The cytosol and mitochondrial fractions were separated via centrifugation at 12,000× *g* for 15 min at 4 °C. The supernatant (cytosol fraction) and mitochondrial pellets were then collected, and the protein concentration was determined via UV fluorescence at 260 nm (UV-2600i spectrophotometer; Shimadzu, Tokyo, Japan). 

To assess the caspase activity in dynamics, cell cultivation was carried out as described above, and the caspases were analyzed after 20, 24, 30, 36 and 48 h of incubation of the cells with **OA** at IC_50_**.** All the detached/dead and viable cells were harvested, and full cell lysates were prepared with RIPA buffer (Cell Signaling Technology, Danvers, MA, USA) with 1.0 mM PMSF as a protease inhibitor (Cell Signaling Technology, Danvers, MA, USA). The cell debris was separated via centrifugation at 14,000× *g* for 10 min at 4 °C. The supernatants were then collected, and the protein concentration was determined using the Lowry method [32]. 

Then, the same protein amounts (20.0 µg in each line) were loaded and separated with 4–20% Mini-PROTEAN TGX Stain-Free Protein Gels (Bio-Rad Laboratories, Hercules, CA, USA), followed by transferring onto polyvinylidene difluoride (PVDF) membranes. The membranes were blocked with 1% (*w*/*v*) casein in Tris-buffered saline containing 0.1% Tween-20 (TBS-T) and were then incubated with primary antibodies (1:1000) of Cytochrome *c* and β-actin (Cell Signaling Technology, Danvers, MA, USA) for cytosol, and mitochondrial fractions, cleavage caspase-3,-6,-7,-8,-9, PARP and β-actin (Cell Signaling Technology, Danvers, MA, USA) for full cell lysates at 4 °C overnight. On the next day, the PVDF membranes were washed thrice in TBS-T and incubated with HRP-conjugated secondary antibodies (1:1000) (Cell Signaling Technology, Danvers, MA, USA) for 1 h at RT. Immunoreactive proteins were detected with Clarity Western ECL Substrate (Bio-Rad Laboratories, Hercules, CA, USA) and were then analyzed on the ChemiDoc MP Imaging system (Bio-Rad Laboratories, Hercules, CA, USA). The proteins were quantified via densitometry using Image Lab software (version 6.0; Bio-Rad Laboratories, Hercules, CA, USA). To normalize protein expression data, a blot with β-actin was used. 

### 2.16. Statistical Analysis 

All the experiments were performed in triplicate. The results are presented as Mean ± SD values. The differences were considered statistically significant at *p* < 0.05. For statistical analysis and graph design, GraphPad Prism 5.0 was used. The statistical significance was evaluated with an unpaired t-test and the Mann–Whitney test.

Calculations of IC_50_ were performed in the “Program for the (half-minimal inhibitory concentration of a substance) chemotherapeutic substances” written in the Laboratory of tumor cell genetics of the N.N. Blokhin National Medical Research Center of oncology of the Health Ministry of Russia (Moscow, Russia).

## 3. Results

### 3.1. The Cytotoxic Effect of **OA** against Human Cancer and Non-Cancerous Cell Lines

**Figure 1:**
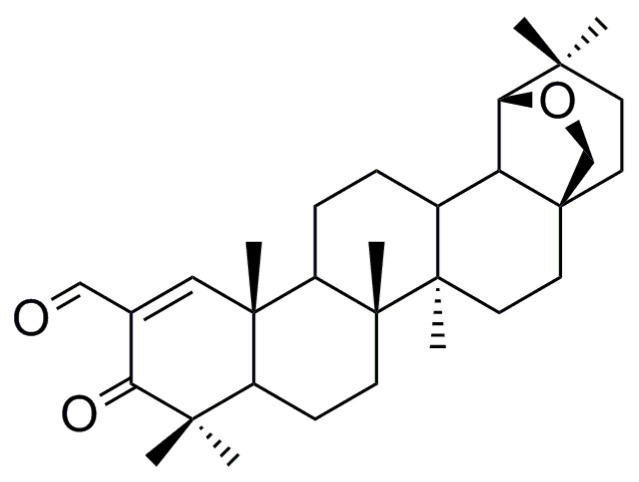
Chemical structure of semi-synthetic triterpenoid **OA**.

We previously described the synthesis of a triterpenic derivative **OA** with high cytotoxicity (IC_50_ 0.66–2.35 μM) against seven cancer cell lines (Hep-2, HCT 116, MS, RD TE32, A549, MCF-7 and PC-3) [21]. The structure of **OA** is presented in Figure 1. 

The new compound with potential anticancer activity should be toxic against the cancer cells, with minimal damage being caused to normal human cells. In order to evaluate the selectivity of **OA** against cancer cells, the cytotoxicity of **OA** against HBL-100 and K562 cell lines and their Dox-resistant subclones was compared with its action against the non-cancerous human breast epithelial cell line MCF10A. The results are shown in Table 1.

As is apparent from Table 1, the tested cancer cells were susceptible to the toxic action of **OA**. In contrast to Dox, **OA** was 6.5–17.8 times more toxic to human cancer cells than it was to non-cancerous MCF10A cells. Thus, **OA** showed certain selectivity against the cancer cells.

It should be noted that HBL-100/Dox and K562/iS9_Dox cells were obtained as a result of the continuous selection of HBL-100 and K562/iS9 cells in the presence of Dox, and K562/iS9 cells originated from K562 cells transfected with the *ABCB1* (*MDR1*) gene [22]. As seen in Table 1, HBL-100/Dox was 105 times more resistant to Dox than the parental HBL-100 cell line (*p* = 0.001) and only 2.3 times more resistant to the toxic effect of **OA** (*p* = 0.01). K562/i-S9 cells were 11 times more resistant to Dox than K562 cells (*p* = 0.0002), and K562/i-S9_Dox cells were 28 times more resistant to Dox than K562 cells (*p* < 0.0001) and 2.6 times more resistant to Dox than K562/i-S9 cells (*p* = 0.0002). Moreover, no statistical difference in IC_50_ of **OA** for the parental K562 cells and resistant subclones was found. K562 vs. K562/i-S9 (*p* = 0.55), K562 vs. K562/i-S9_Dox (*p* = 0.75), K562/i-S9 vs. K562/i-S9_Dox (*p* = 0.73).

The drug resistance of cancer cells is often attributed to the activity of P-gp, the protein participating in the efflux of drugs from the cells. High P-gp expression was demonstrated in resistant subclones of HBL-100/Dox, K562/i-S9 and K562/i-S9_Dox [19,23]. Briefly, P-gp expression in parental HBL-100 and K562 cell lines was negligible, and P-gp-positive cells were practically absent in parental cell lines. P-gp-positive cells accounted for 100%, 80.0 ± 8.2% and 94.7 ± 2.3% of HBL-100/Dox, K562/i-S9 and K562/iS9_Dox-resistant subclones, respectively. Thus, it can be suggested that the resistance of HBL-100/Dox, K562/i-S9 and K562/iS9_Dox cells toward Dox is a result of P-gp activation.

### 3.2. Evaluation of **OA** as a P-Gp Substrate and P-Gp Inhibitor

#### 3.2.1. Molecular Docking with P-Gp

The substances could overcome P-gp-dependent drug resistance due to not being P-gp substrates, and thus, P-gp could not transport them from the cells. On the other hand, the interaction of a substance with P-gp could disturb the P-gp-structure, followed by impairing the transport capability of this protein. Therefore, the FDA recommends that new cytotoxic compounds be tested as P-gp substrates and inhibitors [33]. Many computer models based on the structural similarity of ligands have been developed to predict whether the developed anticancer agents are either P-gp substrates or inhibitors [34]. The web-based analysis confirmed (Table S2) that compound **OA** and well-known P-gp inhibitor verapamil can be classified as P-gp inhibitors. Moreover, **OA**, in accordance with the protocols, was more often predicted as not being a P-gp substrate, in contrast to verapamil. A detailed analysis of individual molecular properties that identified hydrophobicity (MlogP) as a key descriptor for identifying P-gp inhibitors [35] suggested that the hydrophobic triterpenoid **OA** (MlogP 6.79) most likely acts as an inhibitor of the P-gp transporter.

Keeping in mind that the successful interaction of a compound with P-gp can be complicated by space limitations, we performed the docking of compound **OA** into the binding cavity of P-gp as a substrate or as an inhibitor. Because the binding pocket of P-gp is large enough to have multi-binding sites and multi-drug specificities, we performed statistical analysis with 400 docking poses with GOLD 5.0 (CCDC Software), as well as with the best docking pose to find the residues localized in binding pocket. 

**Figure 2:**
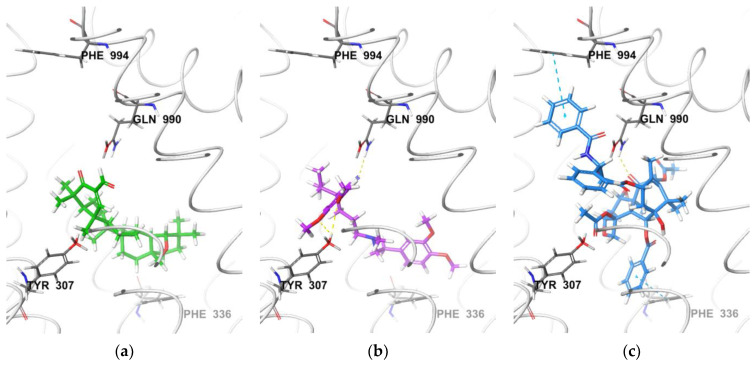
Comparison of the best GOLD docking conformations with human P-gp (PDB ID: 6QEX) for (**a**) **OA**; (**b**) verapamil; and (**c**) co-crystallized taxol.

First, molecular docking with reconstituted human P-gp (PDB ID: 6QEX) was used to calculate whether the compound can act as a P-gp substrate. We found that **OA** can occupy the binding cavity of P-gp similarly to the known substrates (verapamil and co-crystallized taxol (chemical name: paclitaxel)) (Figure 2). However, the highest values of the Chemscore parameter for **OA** did not exceed those for verapamil and taxol (38.16, 40.97 and 43.93, respectively). The analysis of intermolecular interactions of the studied compounds with amino acid residues within 5Å from the binding site showed that **OA**, unlike the known substrates, did not form hydrogen bonds within the binding cavity (Figure 2a), whereas verapamil and taxol formed H-bonds with Tyr307 and Gln990 residues (Figure 2b,c). In addition, taxol formed a Pi–Pi bond with Phe336 and Phe994 residues (Figure 2c). 

**Figure 3:**
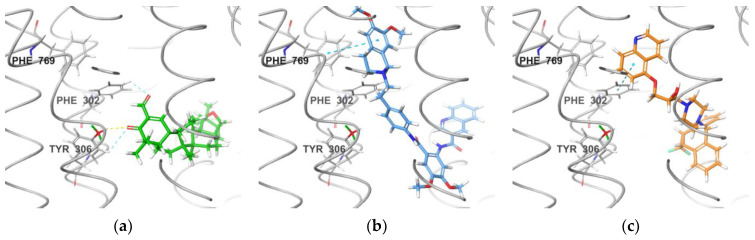
Comparison of the best GOLD docking conformations with human P-gp (PDB ID: 6QEE) for (**a**) **OA**; (**b**) tariquidar; and (**c**) co-crystallized zosuquidar. Dotted line denotes Pi–pi stacking.

Figure 3 illustrates the P-gp structure used in the docking studies with the bound inhibitor zosuquidar and **OA**. For comparison, tariquidar, as a well-known P-gp inhibitor [36], was also docked. As seen in Figure 3, **OA** and tariquidar could occupy the binding cavity of P-gp, simultaneously overlaying the structure of zosuquidar. However, the highest Chemscore values for **OA** did not exceed those for tariquidar and zosuquidar (46.71, 58.49 and 55.00, respectively). **OA** formed two H-bonds with Tyr302 and Tyr306. Strong Pi‒pi interactions were also observed for tariquidar and zosuquidar with Phe769 and Phe302 residues, respectively. Thus, the docking suggested that **OA** is unlikely to be a substrate or inhibitor of P-gp. 

#### 3.2.2. The Effect of OA on Rhodamine 123 Efflux from K562/iS9_Dox Cells

To determine how **OA** overcomes drug resistance induced by P-gp overexpression in cells, we experimentally investigated the influence of this compound on P-gp efflux activity. Rhodamine 123 (Rh123) is a well-known substrate of P-gp, as the transport of Rh123 from cells measured with its fluorescence can indicate the level of P-gp activity. Rh123 efflux from cells could be decreased by another P-gp substrate competing with Rh123 for the binding site of this protein or as a result of P-gp interactions with the compound inhibitor that impairs its structure. 

**Figure 4:**
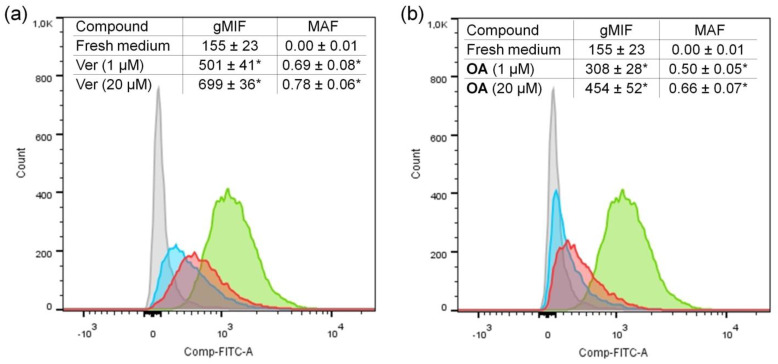
The effect of (**a**) verapamil and (**b**) **OA** on Rh123 efflux from K562/iS9_Dox resistant cells. After 20 min staining with Rh123, the cells were further incubated for 30 min in fresh medium, medium with verapamil or **OA**. Cell luminosity after incubation with Rh123 (green line) followed by incubation in fresh medium (grey area) (**a**) in medium with 1.0 μM (blue line) or 20.0 μM (red line) verapamil, and (**b**) in medium with 1.0 μM (blue line) or 20.0 μM (red line) **OA**. * Statistical significance of gene expression values as compared to fresh medium (*p* < 0.05).

We studied the potential effect of **OA** on Rh123 transport by P-gp to the outside of K562/iS9_Dox cells, with the subclone of K562 cells expressing the largest amount of P-gp. Verapamil, a well-known competitive transport inhibitor of P-gp, was used as a positive control. Concentrations of 1.0 μM (a concentration equal to 2 × IC_50_ of **OA** for K562/iS9_Dox cells) and 20.0 μM (a standard concentration for verapamil in such experiments) were used in our experiments. The results are presented in Figure 4.

After 30 min of incubating the cells stained with Rh123 in a fresh medium (control), the majority of Rh123 was released from the cells, and the gMIF value was only 155 ± 23. Verapamil competed with Rh123 for P-gp binding, diminishing the Rh123 efflux from the cells both at 1.0 and 20.0 μM concentrations (MAF was 0.69 and 0.78, respectively) (Figure 4a). **OA** substantially decreased Rh123 efflux from the cells when used at a highly toxic concentration of 20.0 μM (gMIF value of 454 ± 52 vs. 155 ± 23 for control), but the effect of the 1.0 μM concentration of this substance, which was also rather toxic for these cells, was smaller (gMIF value of 308 ± 28 vs. 155 ± 23 for control) (Figure 4b). Therefore, it seems unlikely that **OA** strongly interacts with P-gp. The concentrations of **OA** used in these experiments—exceeding the IC_50_ value for resistant K562/iS9_Dox cells—indicated that **OA** overcomes cell resistance, not owing to interactions with P-gp. The fact that the addition of **OA** failed to lead to Rh-123 accumulation inside the cells suggests that **OA** is neither a direct inhibitor nor a competitive substrate of this efflux pump.

#### 3.2.3. The Combined Treatment of Cancer Cells with Dox and OA 

In order to determine whether **OA** can modify the toxic action of Dox against resistant cells, a series of MTT assays was carried out using combinations of subtoxic concentrations of OA with different concentrations of Dox. By analyzing the obtained data (Table 2), it could be concluded that adding **OA** at subtoxic concentrations to Dox did not change the toxic effect of Dox against HBL-100, HBL-100/Dox or K562 cells, which confirms the almost unchanged IC_50_ values for Dox. The unchanged IC_50_ values for Dox in resistant subclones confirm that **OA** is not a P-gp inhibitor.

#### 3.2.4. The Effect of OA on ABC Transporter Gene Expression

**Figure 5:**
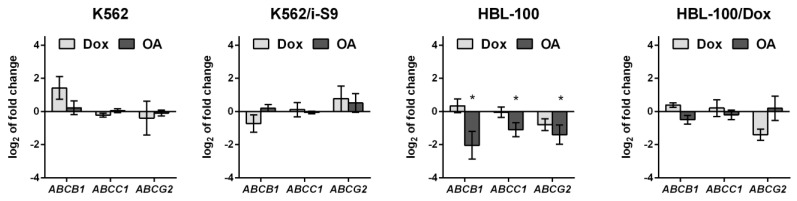
Levels of ABC transporter gene expression (RT-PCR) in K562 and HBL-100 cells and their resistant subclones, K562/iS-9 and HBL-100/Dox cells. The cells were treated with Dox and **OA** at corresponding IC_50_ for 48 h. * Statistical significance of gene expression values as compared to control value (*p* < 0.05).

The possible effect of **OA** on ABC transporter gene expression (*ABCB1*, *ABCC1* and *ABCG2*) in HBL-100, K562 and the resistant subclones of these cells was studied and compared with that of Dox. Concentrations of **OA** and Dox approximately corresponding to their IC_50_ values were used. **OA** had no statistically significant effect on ABC transporter gene expression in K562 or K562/iS-9 cells (Figure 5). In HBL-100 cells, incubation with **OA** resulted in a 2–3-fold decrease in the expression of all ABC transporter genes (*p* < 0.05), but in HBL-100/Dox cells, only a tendency toward decreases in *ABCB1* expression was noted (*p* = 0.1).

### 3.3. **OA** Induces Apoptosis in Parental and Dox-Resistant Cells

#### 3.3.1. The Effect of OA on the Annexin V-FITC/PI Double Staining of HBL-100 and HBL-100/Dox Cells

**Figure 6:**
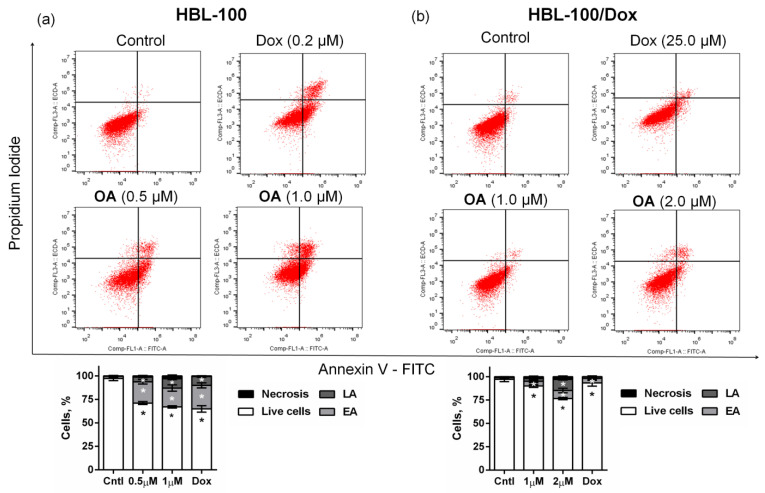
Annexin V-FITC/PI double staining for detection of apoptosis after treatment with OA for 16 h: (**a**) HBL-100; (**b**) HBL-100/Dox cells. LA—late apoptosis; EA—early apoptosis. * Statistical significance of number of cells as compared to control value (*p* < 0.05).

Considering that the main mechanism of the anticancer action of triterpenoids is most often associated with the activation of apoptosis in cancer cells [12,14], the possibility of **OA** inducing apoptotic cell death in adherent HBL-100 and suspension K562 cells and their resistant subclones HBL-100/Dox and K562/iS9 was further analyzed via flow cytometry using Annexin V-FITC/PI double staining (Figure 6 and Figure S1). 

The HBL-100 and HBL-100/Dox cells were treated with **OA** at concentrations close to the IC_50_ and 2 × IC_50_ values (0.5/1.0 and 1.0/2.0 µM, respectively) for 16 h. The results showed **OA** to induce the deaths of both parental and resistant cells through apoptosis (Figure 6). The processing of the Annexin V-FITC/PI staining findings demonstrated an increase in the percentage of early apoptotic (EA) cells from 2.15% to 22.6 and 20.10% for parental cells, and a dose-dependent increase from 1.76% to 5.06 and 8.55% for resistant cells as compared with the control. The content of the late apoptotic (LA) cells was also dose-dependent and tended toward increasing, as compared with the untreated cell culture (from 0.36% to 5.03 and 9.91% for parental cells and from 0.8% to 2.98 and 11.90% for resistant cells). The above-mentioned trend was observed after the action of **OA** on K562 and K562/iS9 suspension cells (Figure S1). Moreover, after incubation with Dox, a characteristic difference was observed for Dox-sensitive and Dox-resistant cancer cells, when the content of early and late apoptotic populations of sensitive cells equaled 25.00 and 9.45%, whereas for resistant cells, these values were significantly lower—4.88 and 1.57%, respectively. Thus, **OA** induced the apoptotic death of both adherent and suspension human cancer cells, as well as their resistant variants, and all types of cells were characterized by significant positive Annexin V staining. The following experiments were carried out for a more detailed assessment of the mechanism of apoptotic cell death upon **OA** treatment using adherent HBL-100 and HBL-100/Dox cells with a normal karyotype.

#### 3.3.2. The Effect of OA on the Cell Cycle of HBL-100 and HBL-100/Dox Cells

**Figure 7:**
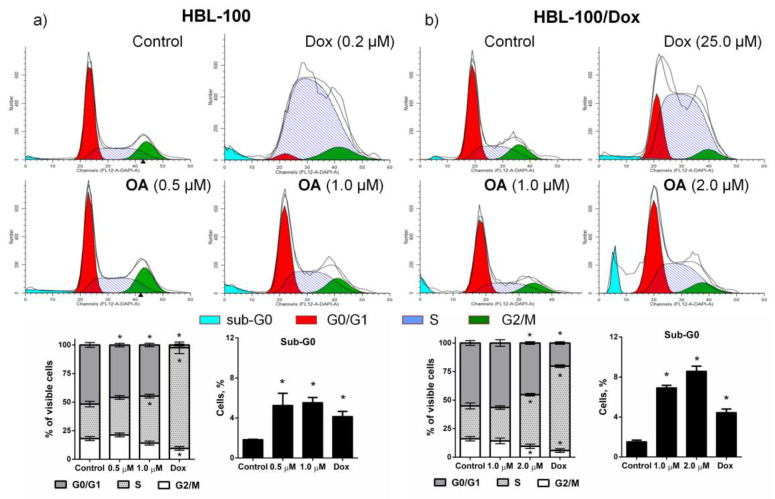
Flow cytometry analysis of the cell cycle phase distribution in cancer cells after treatment with **OA** or Dox for 16 h: (**a**) HBL-100 cells; (**b**) HBL-100/Dox cells. * Statistical significance of number of cells as compared to control value (*p* < 0.05).

The dysregulation of cell death and progression of the cell cycle lead to uncontrolled cell division and, as a result, malignant neoplasms. Therefore, the mechanisms of action of most antitumor drugs are attributed to cell cycle arrest and apoptosis induction. To test the ability of the new compound to regulate the cell cycle, we performed a cell cycle analysis after 16 h of incubation of HBL-100 and HBL-100/Dox cells with **OA** or Dox using flow cytometry and staining with DAPI (Figure 7). In HBL-100 cells incubated in a fresh medium, the following signal distribution was observed: G0/G1 phase—51.76%; S phase—30.12%; and G2/M—18.12%. Incubation of these cells with Dox at a concentration of 0.2 μM resulted in cell cycle arrest in the S phase—88.03% of the HBL-100 cells. No appreciable changes were observed in the cell cycle distribution after the 16 h treatment of the cells with **OA** at 0.5 μM, as follows: G0/G1 phase—45.80%; S phase—32.80%; and G2/M—21.31%. The percentage of the cells in the sub-G0 phase increased from 1.85% in the untreated cells to 5.27 and 5.55% in the cells incubated with 0.5 and 1.0 µM **OA**, respectively. The cell population in the subG0/G1 phase for Dox-treated cells also increased and equaled 4.16%.

In the case of HBL-100/Dox cells, we observed a similar situation for Dox at a concentration of 25.0 μM, which led to cell cycle arrest in the S-phase (73.96%). The **OA** treatment of HBL-100/Dox cells at 2.0 µM led to slight changes in the cell cycle, causing an increase in the proportion of the cells in the S-phase of the cell cycle (45.15% vs. 28.73% for the control). In addition, we observed an appreciable increase in the number of cells in the sub-G0/G1 phase for 1.0 and 2.0 µM **OA** (6.93 and 8.59% vs. 1.54% for the control).

#### 3.3.3. The Effect of OA on DNA Migration and Topoisomerase I Activity

DNA damage is one of the known cellular signals that induce apoptosis. To study the possible ability to interact with DNA, **OA** was incubated for 30 min with the supercoiled plasmide DNA (DNApHOT1, TopoGen) (scDNA) at concentrations from 10.0 μM to 200.0 μM. The anticancer drug carboplatin was used as a positive control. In our study, in contrast to carboplatin, which changed scDNA migration, incubation with **OA**, at concentrations significantly exceeding IC_50_, did not influence scDNA migration, thus indicating that the interaction of **OA** with DNA is unlikely (Figure S2).

Topoisomerases (Topo) modify and regulate the topological state of DNA. They participate in processes such as DNA replication, transcription, recombination and repair [37]. Topo I acts by making a transient single-stranded nick and remaining covalently attached to the 3′-end of the transient break, forming a Topo I cleavage complex. This reaction is reversible, and under normal conditions, only a small fraction of the cleaved DNA is detectable. Topo I inhibitors stabilize cleavage complexes by preventing the DNA regulation step, thus producing irreversible DNA lesions. Topo I unwinds the scDNA to form topoisomers. Therefore, when Topo I is inhibited, a certain part of scDNA remains intact, depending on the degree of inhibition. In our experiments, only the highly cell-toxic concentrations of >20.0 μM **OA** clearly inhibited the activity of Topo I (Figure S3), whereas **OA** concentrations close to IC_50_ only weakly inhibited Topo I. Taking into account that the IC_50_ of **OA** for the human cancer cells tested is less than 2.0 μM, we suggest that **OA** has only a negligible effect on Topo I activity at non-toxic concentrations.

#### 3.3.4. The Effect of OA on p53 Gene

The functionally active nuclear transcription factor p53 responds to upstream signals by activating the transcription of genes that are important for cell cycle arrest, DNA repair and apoptosis [38]. In addition to its transcriptional activity, p53 also induces apoptosis via non-transcriptional mechanisms, including the inhibition of Bcl-2 and Bcl-XL at the mitochondrial membrane [39]. Mutations in the *p53* tumor-suppressor gene are found in over 50% of human tumors, which sometimes display a chemo-resistant phenotype [36]. In this study, the effect of **OA** was investigated by comparing its cytotoxicity against five human cancer cell lines (HCT116, HCT116 p53-/-, MCF-7, MDA-MB-231 and MDA-MB-453) with different p53 statuses (Table 3).

As is apparent from Table 3, the IC_50_ ratio (IC_50_ against HCT116 p53^−/−^ to IC_50_ against HCT 116 wt) clearly shows that p53 knockout HCT116 cells were similarly sensitive to **OA** as *p53* wild-type HCT 116 cells (0.72 vs. 0.76 μM), suggesting that the cytotoxic effect of **OA** (in contrast to Dox) is independent of the p53 status of the cell lines. Moreover, **OA** was equally highly cytotoxic toward three breast cancer cell lines (1.4–2.2 μM), and there was no clear association observed with the p53 statuses of the cell lines.

#### 3.3.5. Confocal Fluorescence Microscopy

Cell apoptosis is tightly associated with chromatin condensation, the loss of mitochondrial transmembrane potential and ROS formation. To clarify the mechanism of apoptosis in HBL-100 and HBL-100/Dox cells after 16 h of **OA** treatment, cellular morphology, mitochondrial permeability and ROS formation were analyzed via confocal fluorescence microscopy using Hoechst 33342, TMRE and H_2_DCFDA staining, respectively. 

**Figure 8:**
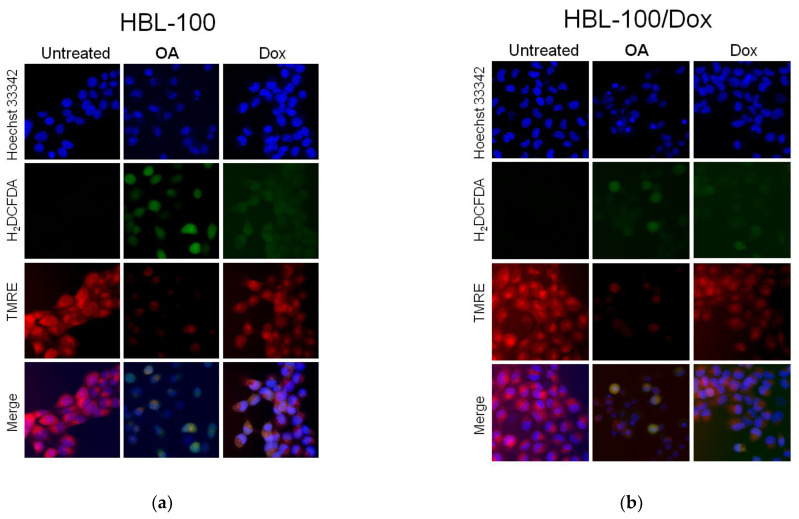
Merged fluorescent images of HBL-100 (**a**) and HBL-100/Dox (**b**) cells stained with Hoechst 33342 (nuclei), TMRE (mitochondria) and H_2_DCFDA (ROS formation) after treatment with **OA** or Dox at IC_50_ for 16 h.

As shown in Figure 8, the untreated HBL-100 and HBL-100/Dox cells had typical oval nuclei with non-condensed chromatin visualized with the DNA-specific dye Hoechst 33342. Despite the fact that all potentiometric dyes are good substrates for MDR carriers, which remove the dye from cells and lead to a decrease in its accumulation [40], we observed a high intensity of red fluorescence in the untreated HBL-100 and HBL-100/Dox cells stained with TMRE. Moreover, we failed to detect any green fluorescence of the native cells after staining with H_2_DCFDA, which measured hydroxyl, peroxyl and other ROS activity within the cell. 

The staining pattern of the Hoechst 33342 dye revealed that **OA**-treated cells contained condensed or fragmented nuclei as indicators of apoptosis. Furthermore, the incubation of HBL-100 and HBL-100/Dox cells with **OA** (0.5 and 1.0 µM, respectively) was also characterized by a significant decrease in mitochondrial fluorescence (ΔΨ_m_ loss), indicating their inability to load the positively charged mitochondrial TMRE indicator. Moreover, **OA** caused ROS formation in HBL-100 and HBL-100/Dox cells, as indicated bythe high intensity of green fluorescence as compared with the control after staining with H_2_DCFDA, whereas the Dox treatment of HBL-100 and HBL-100/Dox cells caused only the wrinkling of nuclei and the light depolarization of the mitochondrial membrane and ROS activity. 

#### 3.3.6. Proteins Responsible for the Induction of Apoptosis

**Figure 9:**
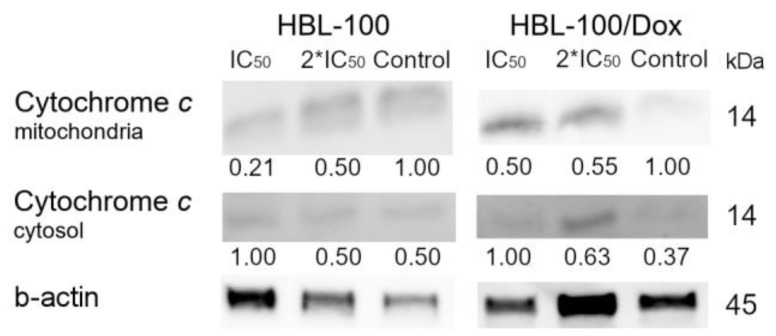
Western blot analysis of the expression of cytochrome *c* in HBL-100 and HBL-100/Dox cells after **OA** treatment for 16 h. β-Actin was used as loading control.

Based on the results that **OA** led to mitochondrial outer membrane permeabilization (Δ_ΨM_), we assessed cytochrome *c* migration following **OA** treatment. Figure 9 evinces the release of cytochrome *c* from mitochondria to cytosol as being more of note in HBL-100/Dox cells. 

**Figure 10:**
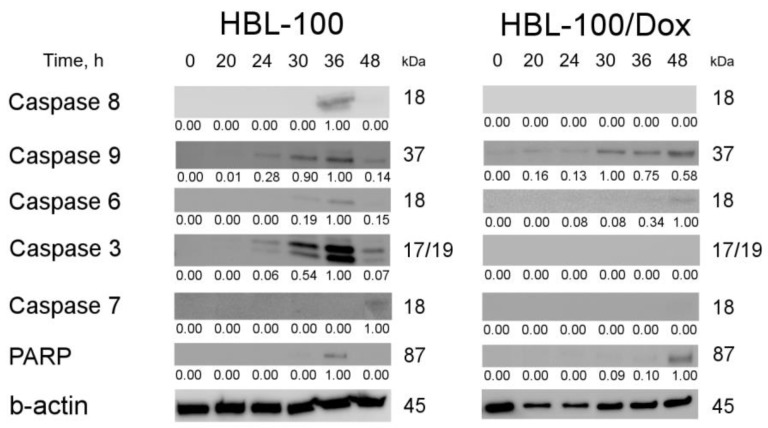
Western blot analysis of the expression of caspases-3,-6,-7,-8, and -9 and PARP in HBL-100 and HBL-100/Dox cells after **OA**-treatment for 20, 24, 30, 36 and 48 h. β-Actin was used as loading control.

Next, we identified whether **OA**-induced apoptosis was associated with the activation of the caspase cascade, and the results showed that **OA** increased caspases-3,-6,-7,-8 and -9 activities and cleaved-poly-ADP ribose polymerase (PARP) (Figure 10). 

The activation of caspase-6,-8 and PARP was recorded in HBL-100 cells after 30–36 h of **OA** treatment, whereas caspase-7 activity was observed only after 48 h of incubation with **OA**. We also observed time-dependent activation of caspases-3,-9 starting with 24 h of incubation with **OA**.

Western blot analysis of HBL-100/Dox cell lysates revealed the expression of cleaved caspase-9 to be time-dependently stimulated by **OA,** starting with 30 h of incubation. Cleaved-PARP and caspase-6 appeared in HBL-100/Dox cells after 48 h of **OA** treatment, whereas caspase-3,-7,-8 activity did not differ from that in the control. 

## 4. Discussion

The toxicity of the triterpenic derivative **OA** with pronounced cytotoxic activity against cancer cells of various origins [21] was additionally studied against breast cancer cells HBL-100 and leukemia cells K562, as well as against their Dox-resistant subclones HBL-100/Dox, K562/i-S9 and K562/iS9_Dox. As is apparent from Table 2, **OA** confirmed its anticancer potential against the cancer cell lines. Concurrently, **OA** showed equal toxicity toward parental HBL-100 and K562 cells and their Dox-resistant subclones. As compared with the non-cancerous human breast epithelial cell line MCF10A, the compound **OA** was 6.5–17.8 times more toxic toward the tested cancer cells. Therefore, we must conclude that **OA** is relatively selective against cancer cells. 

We previously showed that, in the resistant HBL-100/Dox, K562/i-S9 and K562/iS9_Dox cells, the expression of P-gp, the transmembrane protein participating in the efflux of drugs from the cells, was elevated [20,24]. Several possibilities could exist to overcome P-gp-dependent cell resistance under **OA** action. The compound could interact with P-gp in such a way that the native structure of this protein is disturbed, and its transporting function is damaged [7,41]. On the other hand, if a compound is not a P-gp substrate, then it is not able to be transported by a protein out of the cells, which enables this compound to exert its toxic effect independently of cellular P-gp activity.

Numerous P-gp classification structure–activity relationship models based on structurally similar ligands have been developed to predict whether the developed anticancer agents are P-gp substrates or inhibitors [34,42]. In silico approaches are usually swift, inexpensive, less labor-intensive and less time-consuming for drug discovery/prediction and ADME/Tox profiling comparing to in vitro and in vivo assays [43]. The performed analysis based on four available web services [26,27,28,29] enabled us to infer that **OA** unlikely belongs to P-gp substrates (Table S1). 

Because P-gp can undergo substantial conformational changes upon binding with various ligands and has multiple substrate binding sites [44], it is highly challenging to accurately model P-gp–substrate interactions [45,46,47]. The recently available cryo-electron microscopy structure of the human–mouse chimeric P-gp in a complex with substrates and inhibitors [48] avoids the use of homologous modeling that we previously used [20]. Molecular docking with the human–mouse chimeric P-gp revealed that **OA** may occupy a part of the transmembrane-binding cavity of P-gp but not as successfully as known substrates verapamil or PTX do, without hydrogen bond formation within the binding cavity (Figure 3). Interestingly, another betulin derivative, betulinic acid (BA), was also determined as not being a substrate of any of the three ABC transporters (MDR1/ABCB1, ABCG2 and ABCB5), but the cancer cells overexpressing these transporters were efficiently responded to by BA [18]. 

Docking studies have also shown the **OA** binding site to overlap with the binding sites of the known P-gp inhibitors tariquidar and zosuquidar in the transmembrane domain of the P-gp molecule [36]. However, it is unlikely that **OA** acts as an inhibitor due to its inability to form strong bonds within the P-gp binding pocket, similar to tariquidar and zosuquidar (Figure 3). Most likely, a strong bond with P-gp and, as a result, high P-gp inhibitory activity are provided by the presence of aromatic rings in the tariquidar and zosuquidar structures, which are capable of forming Pi–pi staking with Phe302 and Phe769 residues of the protein.

It was advisable to check whether **OA** can interact with P-gp directly. For this purpose, we evaluated its capability to inhibit the efflux of the well-known P-gp substrate Rh123 from K562/iS9_Dox, the cells with a high level of P-gp expression. Incubation of the cells with Rh123, together with a typical P-gp substrate, verapamil, resulted in competitive inhibition and a dose-dependent decrease in the release of Rh123, as assessed by its fluorescence. As can be seen from Figure 4, only the highly toxic concentration of **OA** caused the minor inhibition of Rh123 efflux from K562/iS9_Dox cells. In turn, the **OA** concentrations that were close to the IC_50_ had a negligible effect on Rh123 efflux from cells, thus indicating that **OA** is most likely not a substrate of P-gp at the IC_50_. According to Kannan et al. [49], the overall effect of inhibitors on the function of transporters, like the action of enzymes and receptors, depends on the relative concentrations of the inhibitor and its target protein; for example, at low concentrations, tariquidar acted as a P-gp inhibitor as well as a substrate of BCRP, whereas at much higher concentrations (≥100 nM), it acted as an inhibitor of both P-gp and BCRP. 

The ability of pentacyclic triterpenoids to sensitize the susceptibility of human cancer cells to chemical drugs was recently shown [50]. In our previous study, the addition of semi-synthetic triterpenoids at subtoxic concentrations significantly increased the sensitivity of Dox-resistant HBL-100/Dox and K562/iS9 cells to Dox action, indicating a synergistic effect of triterpenoids with Dox against MDR cells [20]. A similar effect of an **OA** structural analog was previously found with the semi-synthetic triterpenoid CDDO-Me, which passed phase I/II clinical trials for cancer treatment [51] and which induced an antiproliferative effect in Pgp1, over-expressing KHOS_R2_ and U-2OS_TR_ cells synergistically with Dox [52]. Therefore, we also investigated the combined action of Dox and **OA** on cell viability in HBL-100, HBL-100/Dox, K562 and K562/iS9_Dox cells, but no effect of the subtoxic concentrations of **OA** on Dox toxicity was found. Our findings confirm the suggestion that **OA** cannot successfully interact directly with P-gp. The assumption that **OA** is not a substrate of P-gp leads to an inference that **OA** is not released from HBL-100/Dox cells and is able to accumulate in the intracellular space.

In contrast to CDDO-Me, which had no genotoxic effect on Pgp1 expression [52], the triterpenic derivatives previously synthesized by us suppressed not only the transport function of the P-gp protein but also the expression of ABC transporter genes in MDR cells [19]. Therefore, it was logical to study the **OA** effect on ABC transporter gene expression in HBL-100, HBL-100/Dox, K562 and K562/iS9_Dox cells. P-gp is encoded by the *ABCB1* (*MDR1*) gene, and the *ABCC1* (*MRP1*) and *ABCG2* (*BCRP*) genes encode two other members of ABC transporters. **OA** had no statistically significant effect on ABC transporter gene expression in K562, K562/iS9_Dox and HBL-100/Dox cells (Figure 5). In the Dox-resistant HBL-100/Dox culture, there was only a trend toward a decrease in the expression of *ABCB1*. However, in HBL-100 cells, **OA** statistically significantly inhibited the expression of all ABC transporter genes that were studied. It should be noted that incubation with **OA** did not lead to an increase in the expression of ABC transporter genes in any of the cell lines studied, which may indicate that **OA** does not activate drug resistance via the mechanism associated with ABC transporters. 

We then showed that the decrease in viability of both the original K562 and HBL-100 cells and their resistant variants after **OA** treatment was caused by apoptosis with the characteristic externalization of phosphatidylserine, leading to significant positive Annexin V staining (Figure 6 and Figure S1). There was a dose-dependent increase at the levels of total (early and late) apoptotic cells in the **OA**-treated HBL-100 and HBL-100/Dox cells. Thus, **OA** treatment for 16 h caused an increase in the subG0/G1 phase cell cycle in HBL-100 and HBL-100/Dox cells (up to 9%), and the number of HBL-100 cells in the G0/G1, S or G2/M phases did not change as compared with the number of untreated cells. Moreover, **OA** as well as Dox caused an increase in the number of the cell population in the S phase cell cycle for the HBL-100/Dox cell line (Figure 6). 

Apoptosis is associated with cellular DNA damage, which can be both a consequence of apoptosis and its cause [53]. DNA-destructive factors include compounds that directly interact with DNA or those that impair the function of proteins participating in DNA reparation, or the relaxation of supercoiled DNA required during its replication and transcription. DNA damage induces cell cycle arrest through the activation of checkpoints allowing the time for DNA reparation; otherwise, the cell undergoes apoptosis [51]. Therefore, to clarify the mechanism of **OA** toxicity, we investigated the interaction of this compound with DNA and its possible influence on Topo I activity. In our study, no interaction of **OA** with plasmid supercoiled DNA was found (Figure S2). Interestingly, a number of triterpenoids, including the aforementioned BA, are known as potent inhibitors of eukaryotic Topo I and Topo II [54]. Along with that, **OA** clearly inhibited Topo I activity only at highly toxic concentrations exceeding 20.0 μM (Figure S3), and the IC_50_ for the tested cancer cell cultures ranged from 0.45 to 2.20 μM (Table 1 and Table 3). Therefore, the IC_50_ of **OA** is likely to produce a minor effect on Topo I. Therefore, it can be inferred that apoptosis induced by **OA** in parental and resistant cancer cells is not mediated by DNA damage. In turn, the p53 tumor suppressor continues to be distinguished as the most frequently mutated gene in human cancer and plays a major role in the response of cancer cells to DNA damage anticancer substances [36]. However, we did not observe a clear association with *p53* expression (p53-dependent apoptosis) and the cytotoxicity of **OA** by using a panel of five cancer cell lines with different p53 statuses (Table 3). 

The relationship between increased ROS production and the inhibition of P-gp function and the expression of ABC transporter genes in MDR cells has been previously confirmed [55,56]. Moreover, Dox, a potent exogenous ROS generator [57] and ROS hyper generator after Dox treatment with cells or under hypoxic conditions, can lead to the activation of HIF-1α and can cause an increase in the activity and expression of P-gp, promoting Dox resistance [58]. Therefore, we tested the ability of **OA** to induce ROS production in parental and resistant cells. As can be seen from Figure 8, ROS formation after **OA** treatment was more pronounced in HBL-100 and HBL-100/Dox cells than that after Dox-treatment. Kim et al. showed [59] that the ability of CDDO-imidazolide to induce DNA damage in tumor cells is associated with ROS generation. Native betulin and betulonic acid have also been found to directly affect mitochondria and their membranes, inhibiting the activity of the complexes of the respiratory chain of organelles, thereby initiating ROS overproduction and mitochondrial dysfunction [60]. In this study, **OA** disrupted the oxidative balance of cells, but it was difficult to establish whether an increase in cellular ROS was an initiating event, or, for example, if it might be a consequence of mitochondrial perturbation. We showed **OA** to effectively depolarize mitochondrial membranes (Figure 8), the increased permeability of which can lead to the leakage of the number of pro-apoptotic factors, including cytochrome *c*, thus triggering apoptosis [61,62]. The biological effects described for pentacyclic triterpenoids are partly explained by their structural similarity with sterols, their ability to interact with artificial cholesterol-enriched membrane domains and, therefore, their ability to influence the heterogeneity of the lateral domains of cell membranes [63]. Therefore, BA exhibited a strong ability to interact with and damage synthetic membranes, disturb mitochondrial membranes and destabilize lysosomes [64].

Apoptosis, one of the better-known forms of regulated cell death, is activated when cell surface death receptors, such as Fas, are engaged by their ligands (the extrinsic pathway) or when BCL-2-family pro-apoptotic proteins cause the permeabilization of the mitochondrial outer membrane (the intrinsic pathway) [65]. Both pathways lead to the activation of caspases, a family of cysteine proteases responsible for cell death; they primarily activate caspase-8 (the extrinsic pathway) or caspase-9 (the intrinsic pathway) to further induce caspase-3 activation and subsequently lead to the cleavage of various substrates, such as PARP [66]. As can be seen from Figure 8, Figure 9 and Figure 10, **OA** induced ROS generation, the depletion of MMP (ΔΨ_m_) and cytochrome *c* translocation, and it promoted the activities of caspases in parental and resistant cancer cells. It is of interest that **OA** treatment led to early apoptotic changes in HBL-100 cells, and that these changes were simultaneously associated with extrinsic and intrinsic pathways mediated by the activation of caspases-8,-9,-3,-6,-7. On the basis of the band intensity (Figure 10), it is likely that both pathways, the death receptor and the mitochondrial pathways, synergize and amplify each other’s signals in parental HBL-100 cells. **OA**-induced apoptosis in HBL-100/Dox cells was found to increase only the expression of the caspase-9 protein associated with the intrinsic pathway of cell death (Figure 10). After **OA** treatment, the activation of caspase-6 and the emergence of cleaved-PARP in HBL-100/Dox cells, without caspase-3 activation, were also recorded. The functions of other executioner caspases, such as caspases-6,-7, have been proposed to partially replace caspase-3 activity [67], and, although PARP is primarily a substrate for caspases-3,-7, it is also recognized as a minor substrate for caspase-6 [68]. Similarly, several pentacyclic triterpenoids act as inducers of intrinsic and extrinsic apoptotic signaling pathways. For example, CDDO-Me was shown to activate both extrinsic and intrinsic apoptosis pathways in PC-3 cells [69]. Keto-beta-boswellic acid and acetyl-keto-beta-boswellic acid also induce apoptosis in HT-29 cells through caspase-8, -9 and -3 activation [70]. Although caspase-8 was shown to activate caspase-9 indirectly in type II cells via cleavage of the Bid protein, a member of the bcl-2 family, and the subsequent release of mitochondrial cytochrome *c* [71], there has been no evidence to-date of the direct processing of caspase-9 in vivo by this initiator caspase [72]. In this study, cross communication between the two pathways was unlikely, because caspase-9 was activated after 24 h, and caspase-8 was activated after 36 h of OA treatment.

Thus, parental HBL-100 cells were characterized by **OA**-induced apoptosis with the release of mitochondrial cytochrome *c* into cytosol and by the subsequent activation of the signaling proteins of the extrinsic and intrinsic pathways, accompanied by PARP cleavage, whereas only the mitochondrial-associated intrinsic apoptosis pathway drove cellular apoptosis in resistant HBL-100/Dox cells.

## 5. Conclusions

Overall, this study demonstrates that **OA** is neither an inhibitor nor a substrate of P-gp, and it can overcome P-gp-dependent cell drug resistance and induce apoptosis by provoking ROS generation and mitochondria membrane depolarization upregulating caspase activation. To better understand the multiple mechanisms of **OA** action, further detailed studies of the relationship between ROS generation and apoptosis induction in MDR cancer cells are required.

## Figures and Tables

**Table 1:** Cytotoxicity of **OA** against human cancer cell lines, non-cancerous human breast epithelial cells and resistant subclones (MTT-test, 72 h).

Cell Line		IC_50_, μM (Mean ± SD)	
		Dox	OA
Dox-sensitive cells			
Human mammary epithelial cells immortalized by SV-40 virus	HBL-100	0.24 ± 0.07	0.53 ± 0.04
Chronic myelogenous leukemia	K562	0.36 ± 0.17	0.47 ± 0.13
Dox-resistant subclones			
	HBL-100/Dox	25.20 ± 5.10	1.24 ± 0.19
	K562/i-S9	3.90 ± 1.60	0.45 ± 0.13
	K562/iS9_Dox	10.30 ± 1.20	0.51 ± 0.24
Non-cancerous cells			
Human breast epithelial cell line	MCF10A	0.45 ± 0.1	8.02 ± 0.28

**Table 2:** Cytotoxicity of Dox and combination of Dox and **OA** toward HBL-100 and K562 cells and their resistant subclones. Subtoxic concentrations of **OA** were used (MTT test, 72 h).

Cell Line	IC_50_, μM (Mean ± SD)		Amount of OA Added (μM)
	Dox	Dox (In the OA Presence)	
HBL-100	0.24 ± 0.07	0.24 ± 0.05	0.20
HBL-100/Dox	25.20 ± 5.10	23.30 ± 6.20	0.60
K562	0.36 ± 0.17	0.39 ± 0.02	0.20
K562/i-S9	3.90 ± 1.60	2.00 ± 0.20	0.20

**Table 3:** **OA** sensitivity in panel of cancer cell lines with different p53 statuses (MTT test, 72 h).

**Cell Line**	**p53 Status**	**IC_50_, μM (Mean ± SD)**	
		**Dox**	**OA**
HCT116	wt	1.4 ± 0.6	0.76 ± 0.15
HCT116 p53^−/−^	deletion	3.6 ± 0.5 *	0.72 ± 0.13
MCF7	wt	0.14 ± 0.07	1.93 ± 0.30
MDA-MB-231	mutant	0.34 ± 0.03 *	1.40 ± 0.50
MDA-MB-453	mutant	1.5 ± 0.5 *	2.20 ± 0.24

* Statistical significance of IC_50_ values as compared with IC_50_ value of corresponding wt (*p* < 0.05).

## Data Availability

Data is contained within the article and Supplementary Material.

## Supplementary Materials

The following are available online at https://www.mdpi.com/article/10.3390/biology12030415/s1: Table S1: Primers for real-time PCR; Table S2: Prediction of **OA** and verapamil as P-pg substrate or P-gp inhibitor via web services; Figure S1: Annexin V-FITC/PI double staining for detection of apoptosis in (a) K562 and (b) K562/iS9 cells after treatment with **OA** for 24 h; Figure S2: Interaction of **OA** with supercoiled DNA; Figure S3: Effect of **OA** on the activity of Topoisomerase I; Figure S4: Expression of cytochrome *c* in HBL-100 and HBL-100/Dox cells after **OA** treatment for 16 h, Figure S5: Expression of caspases-3,-6,-7,-8 and -9 and PARP in HBL-100 cells after **OA**-treatment for 20, 24, 30, 36 and 48 h; Figure S6: Expression of caspases-3,-6,-7,-8 and -9 and PARP in HBL-100/Dox cells after OA treatment for 20, 24, 30, 36 and 48 h.
