# Supplementary material for: The Molecular Mechanisms of Oleanane Aldehyde-β-enone Cytotoxicity against Doxorubicin-Resistant Cancer Cells
Source: Biology (Basel). 2023 Mar 8;12(3):415. doi: 10.3390/biology12030415 (PMC10045559; doi:10.3390/biology12030415)
Supplement: Supplementary file 1 [file biology-12-00415-s001.zip › biology-2250758-supplementary.pdf]

Supporting Information

The molecular mechanisms of oleanane aldehyde-β-enone cytotoxicity against doxorubicin-resistant cancer cells

Natalia Moiseeva, Daria Eroshenko, Lidia Laletina, Ekaterina Rybalkina, Olga Susova, Aida Karamysheva, Irina Tolmacheva, Mikhail Nazarov, and Victoria Grishko

Table S1. Primers for Real-Time PCR.

Gene	Forward	Reverse
ABCB1	GGGATGGTCAGTGTTGATGGA	GCTATCGTGGTGGCAAACAATA
ABCC1	GTGAATCGTGGCATCGACATA	GCTTGGGACGGAAGGGAATC
ABCG2	TGAGCCTACAACTGGCTTAGA	CCCTGCTTAGACATCCTTTTCAG
RPL0	CCTTCTCCTTTGGGCTGGTCATCCA	CAGACACTGGCAACATTGCGGACAC

Table S2. Prediction of **OA** and verapamil as P-pg substrate or P-gp inhibitor via web services

Web service	Substrate		Inhibitor	
	**OA**	Verapamil	**OA**	Verapamil
AdmetSAR (version 2.0) https://admetmesh.scbdd.com/	---	+++	---	+++
ADMETlab 2.0 http://lmmd.ecust.edu.cn/admetsar2	-	+	+	+
pkCSM http://biosig.unimelb.edu.au/pkcsm/	-	+	+	+
PgpRules https://pgprules.cmdm.tw/	+	+	+	+

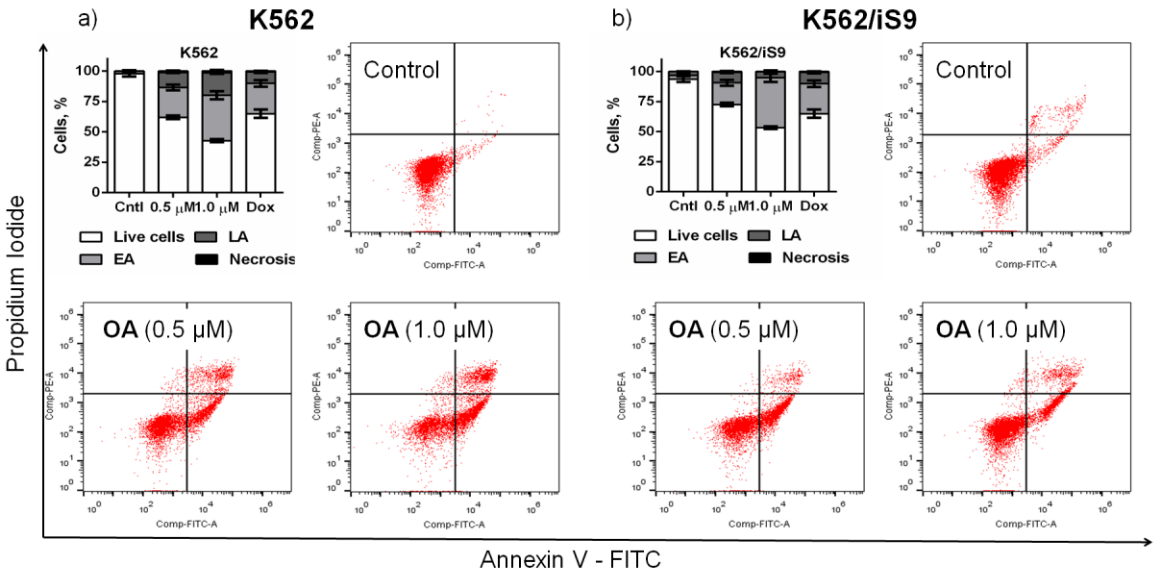

Figure S1. Annexin V-FITC/PI double staining for detection of apoptosis in K562 (a) and K562/iS9 (b) cells after treatment with **OA** for 24 h: LA – late apoptosis, EA – early apoptosis.

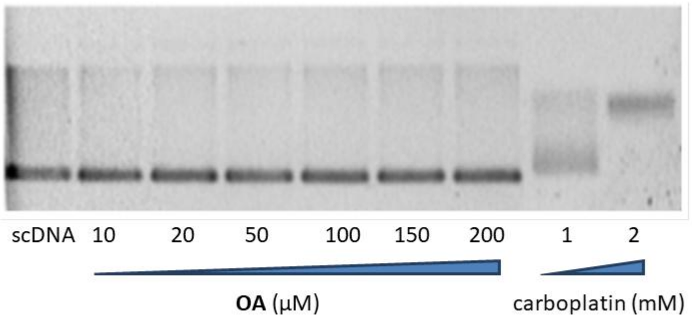

Figure S2. The interaction of **OA** with supercoiled DNA.

The interaction of the substances with DNA causes the retardation of DNA migration in agarose gel-electrophoresis. Carboplatin was used as a positive control. The supercoiled (scDNA) was incubated for 30 min with **OA** or carboplatin.

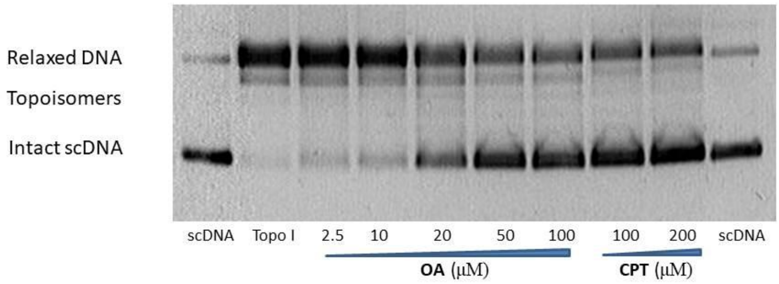

Figure S3. The effect of **OA** on the activity of Topoisomerase I.

Camptothecin (CPT) was used as a positive control.

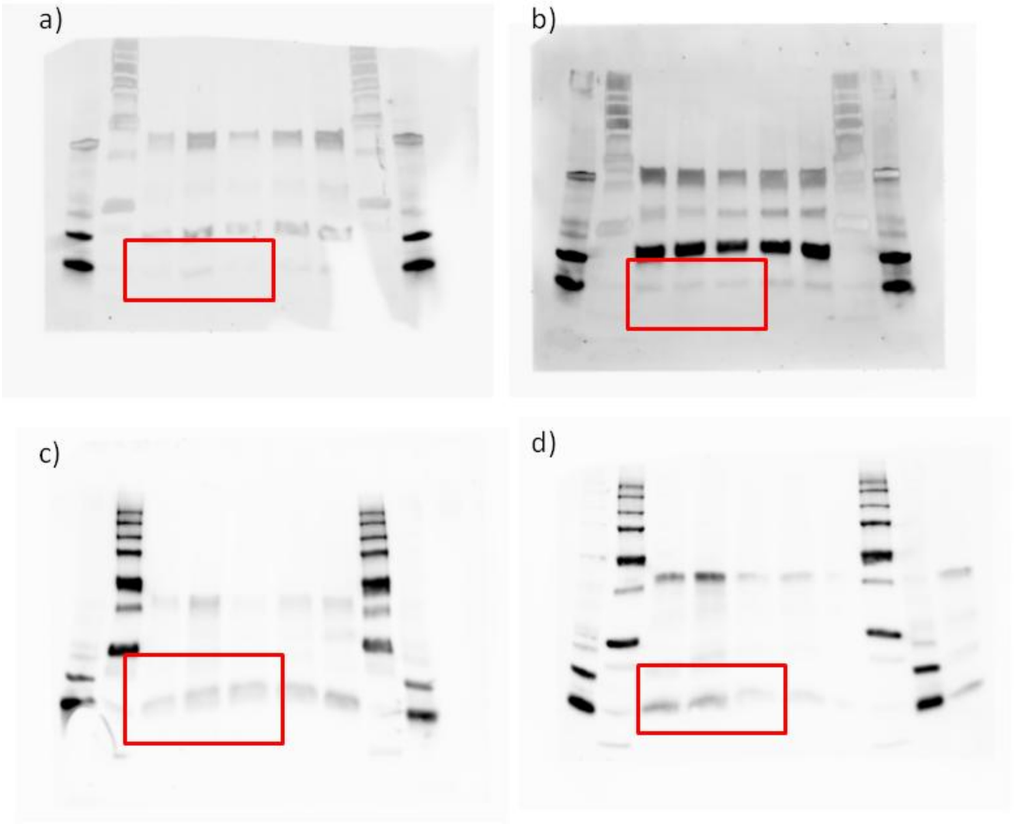

Figure S4: Expression of cytochrome *c* in HBL-100 and HBL-100/Dox cells after **OA**-treatment for 16 h. Red squares indicate lines used in this study

a) WB of cytosolic fraction of the HBL-100 cell lisates after **OA**-treatment for 16 h with 0.5 and 1.0 µM: line 1, 6, 7, 10 – inapplicable in this research, lines 2, 8 - MW, lines 3 - 4 – **OA**-treatment with 0.5 and 1.0 µM, line 5 - control. Staining with primary AT of cytochrom *c* (18 kDa)

b) WB of cytosolic fraction of the HBL-100/Dox cell lisates after **OA**-treatment for 16 h with 1.0 and 2.0 µM: line 1, 6, 7, 10 – inapplicable in this research, lines 2, 8 - MW, lines 3 - 4 – **OA**-treatment with 1.0 and 2.0 µM, line 5 - control. Staining with primary AT of cytochrom *c* (18 kDa)

c) WB of mitochondrial fraction of the HBL-100 cell lisates after **OA**-treatment for 16 h with 0.5 and 1.0 µM: line 1, 6, 7, 10 – inapplicable in this research, lines 2, 8 - MW, lines 3 - 4 – **OA**-treatment with 0.5 and 1.0 µM, line 5 - control. Staining with primary AT of cytochrom *c* (18 kDa)

d) WB of mitochondrial fraction of the HBL-100/Dox cell lisates after **OA**-treatment for 16 h with 1.0 and 2.0 µM: line 1, 6, 7, 10 – inapplicable in this research, lines 2, 8 - MW, lines 3 - 4 – **OA**-treatment with 1.0 and 2.0 µM, line 5 - control. Staining with primary AT of cytochrom *c* (18 kDa)

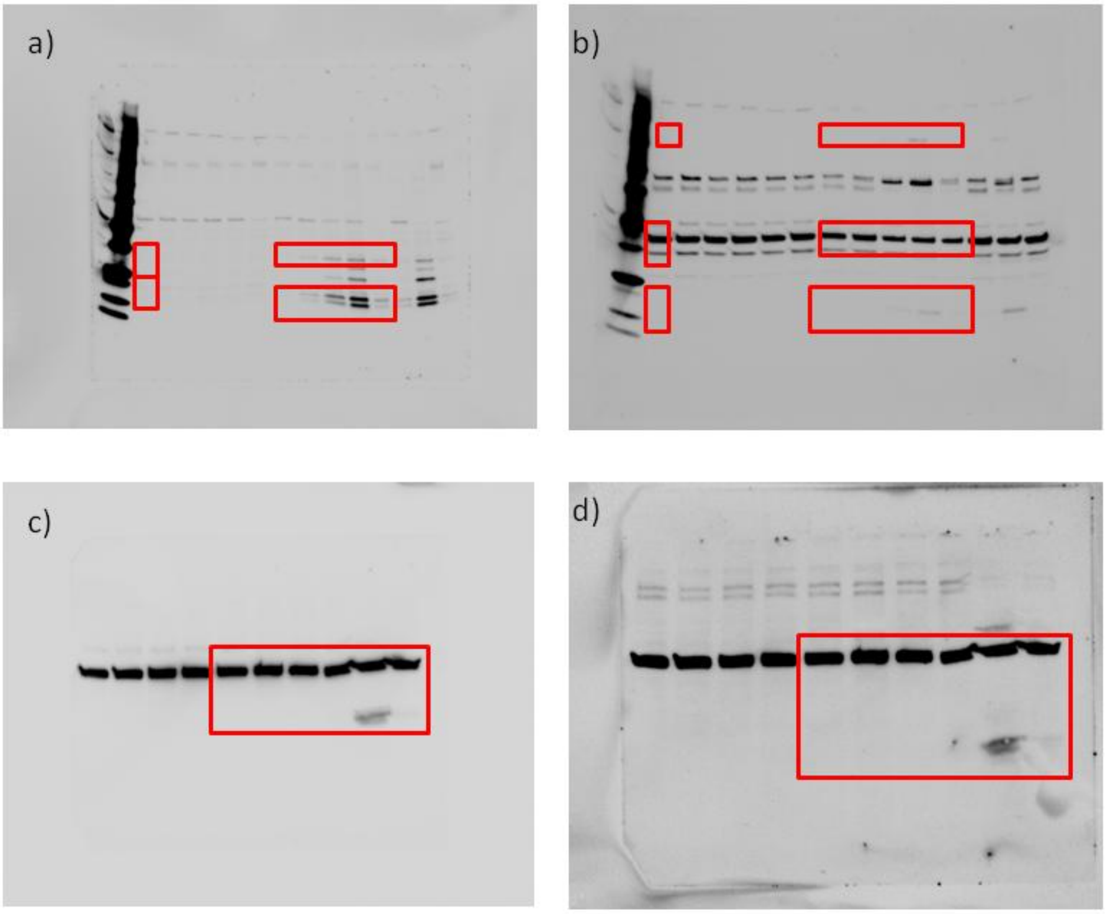

Figure S5: Expression of caspases-3,-6,-7,-8, and -9 and PARP in HBL-100 cells after OA-treatment for 20, 24, 30, 36 and 48 h. Red squares indicate lines used in this study

a) WB of HBL-100 cell lisates after **OA**-treatment for 20, 24, 30, 36, 48 h: line 1 – MW; Line 2 – Control ; lines 3 -7, 13-15 - inapplicable in this research; lines 8 - 12 – **OA**-treatment for 20, 24, 30, 36, 48 h. Staining with primary AT of cleavage caspase-3 (17/19 kDa) and caspase-9 (37 kDa)

b) WB of HBL-100 cell lisates after **OA**-treatment for 20, 24, 30, 36, 48 h: line 1 – MW; Line 2 – Control ; lines 3 -7, 13-15 - inapplicable in this research; lines 8 - 12 – **OA**-treatment for 20, 24, 30, 36, 48 h. Staining with primary AT of cleavage caspase-6 (18 kDa), b-actin (45 kDa) and PARP (89 kDa).

c) WB of HBL-100 cell lisates after **OA**-treatment for 20, 24, 30, 36, 48 h lines 1 - 4 - inapplicable in this research; line 5 – control ; lines 6 - 9 – **OA**-treatment for 20, 24, 30, 36, 48 h. Staining with primary AT of cleavage caspase-8 (18 kDa) and b-actin (45 kDa)

d) WB of HBL-100 cell lisates after **OA**-treatment for 20, 24, 30, 36, 48 h: lines 1 - 4 - inapplicable in this research; line 5 – control ; lines 6 - 9 – **OA**-treatment for 20, 24, 30, 36, 48 h. Staining with primary AT of cleavage caspase-7 (18 kDa) and b-actin (45 kDa)

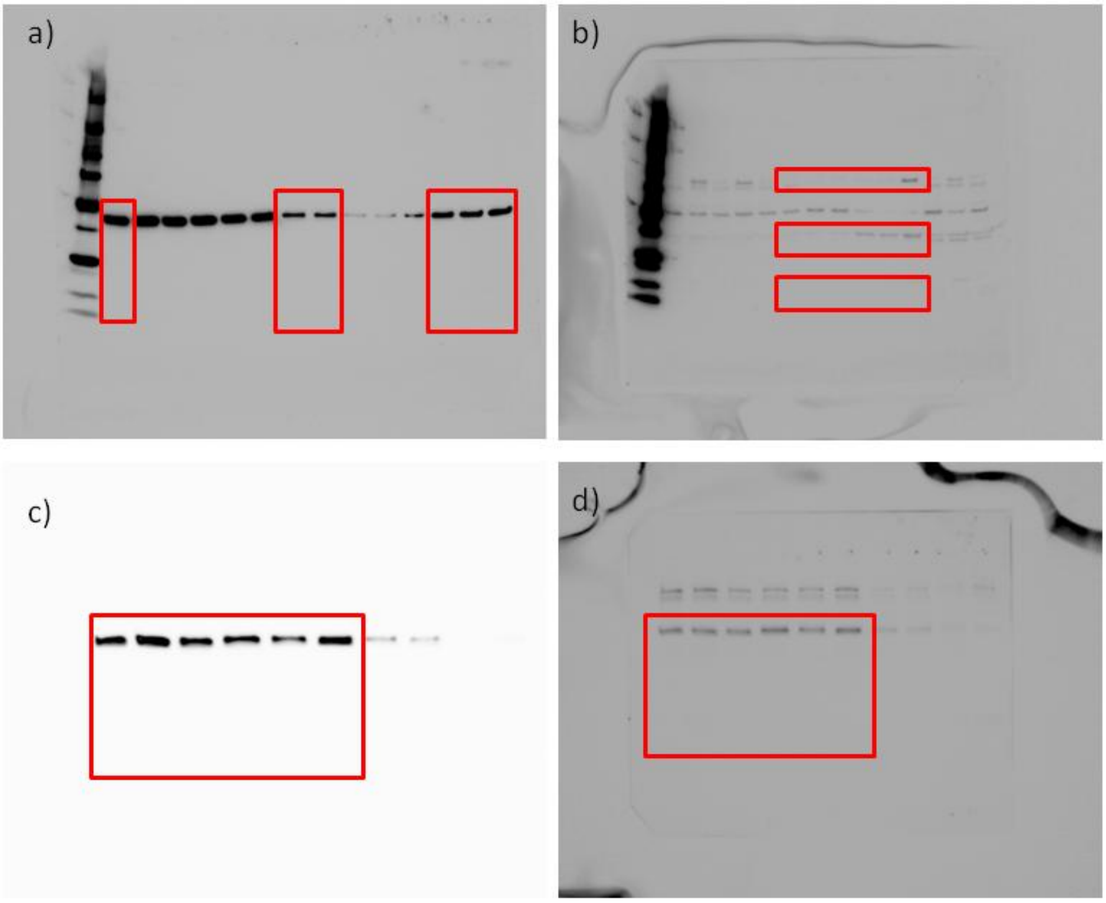

Figure S6: Expression of caspases-3,-6,-7,-8, and -9 and PARP in HBL-100/Dox cells after **OA**-treatment for 20, 24, 30, 36 and 48 h. Red squares indicate lines used in this study

a) WB of HBL-100/Dox cell lisates after **OA**-treatment for 20, 24, 30, 36, 48 h: line 1 – MW; lines 2-6. 10- 12 - inapplicable in this research; line 7 – Control ; lines 8, 9 – **OA**-treatment for 20, 24 h; lines 13-15 – **OA**-treatment for 30, 36, 48 h. Staining with primary AT of cleavage caspase-3 (17/19 kDa) and b-actin (45 kDa)

b) WB of HBL-100/Dox cell lisates after **OA**-treatment for 20, 24, 30, 36, 48 h: line 1 – MW; lines 2-6, 13-15 - inapplicable in this research; line 7 – Control ; lines 8 - 12 – **OA**-treatment for 20, 24 , 30, 36, 48 h**.** Staining with primary AT of cleavage caspase-6 (18 kDa), caspase-9 (37 kDa) and PARP (89 kDa).

c) WB of HBL-100/Dox cell lisates after **OA**-treatment for 20, 24, 30, 36, 48 h: line 1 – Control ; lines 2 -5 - **OA**-treatment for 20, 24, 30, 36, 48 h; lines 6 - 9 – inapplicable in this research. Staining with primary AT of cleavage caspase-8 (18 kDa) and b-actin (45 kDa).

d) WB of HBL-100/Dox cell lisates after **OA**-treatment for 20, 24, 30, 36, 48 h: line 1 – Control ; lines 2 -5 - **OA**-treatment for 20, 24, 30, 36, 48 h; lines 6 - 9 – inapplicable in this research. Staining with primary AT of cleavage caspase-7 (18 kDa) and b-actin (45 kDa)
